# Target control of linear directed networks based on the path cover problem

**DOI:** 10.1038/s41598-024-67442-7

**Published:** 2024-07-23

**Authors:** Wataru Someya, Tatsuya Akutsu, Jose C. Nacher

**Affiliations:** 1 Department of Information Science, Faculty of Science, Toho University, Funabashi, Chiba 274-8510 Japan; 2 Bioinformatics Center, Institute for Chemical Research, Kyoto University, Kyoto, Uji 611-0011 Japan

**Keywords:** Complex networks, Network topology

## Abstract

Securing complete control of complex systems comprised of tens of thousands of interconnected nodes holds immense significance across various fields, spanning from cell biology and brain science to human-engineered systems. However, depending on specific functional requirements, it can be more practical and efficient to focus on a pre-defined subset of nodes for control, a concept known as *target control*. While some methods have been proposed to find the smallest driver node set for target control, they either rely on heuristic approaches based on *k*-walk theory, lacking a guarantee of optimal solutions, or they are overly complex and challenging to implement in real-world networks. To address this challenge, we introduce a simple and elegant algorithm, inspired by the path cover problem, which efficiently identifies the nodes required to control a target node set within polynomial time. To practically apply the algorithm in real-world systems, we have selected several networks in which a specific set of nodes with functional significance can be designated as a target control set. The analysed systems include the complete connectome of the nematode worm *C. elegans*, the recently disclosed connectome of the *Drosophila* larval brain, as well as dozens of genome-wide metabolic networks spanning major plant lineages. The target control analysis shed light on distinctions between neural systems in nematode worms and larval brain insects, particularly concerning the number of nodes necessary to regulate specific functional systems. Furthermore, our analysis uncovers evolutionary trends within plant lineages, notably when examining the proportion of nodes required to control functional pathways.

## Introduction

Network controllability methods have been developed by integrating control theory concepts with traditional network features. This innovative approach potentially enables us to manipulate large-scale systems using only a limited number of nodes as input signals^1–3^. These crucial nodes, connected to external signals, are referred to as *driver nodes*. In the field of structural controllability in complex networks, the primary goal is to identify the minimum set of driver nodes. By controlling the states of these nodes, we are able to guide the states of the remaining nodes toward desired states in finite time.

Liu et al. proposed the Maximum Matching (MM)-based model to address network controllability^1^. They demonstrated that for networks with linear dynamics, determining a minimum set of driver nodes involves computing a maximum bipartite matching^1^. Nacher and Akutsu established a relationship between a Minimum Dominating Set (MDS) and structural controllability for linear and non-linear systems^2^. Both models have been extensively applied to analyse real-world networks. For example, Wuchty conducted an in-depth analysis of controllability in protein–protein interaction networks in human and yeast organisms using the MDS approach. By examining oncogenes and tumor suppressor genes, a significant association with the identified MDS of proteins was observed^4^. Moreover, various algorithms have been devised to identify MDS and classify their control categories, yielding substantial biological insights into disease-related genes and critical control proteins across diverse biological systems^5–10^.

On the other hand, the MM model has also played a key role in uncovering associations between control nodes and biological characteristics like essential and cancer genes in protein interaction networks^1,11^. Mutations in driver genes linked to these nodes are thought to drive the transition from normal to diseased states. Moreover, the MM model has provided insights into specific neuron functions in nematode neuronal circuitry through combined ablation experiments and controllability analysis^12^. Additionally, the MM model has been applied to understand the control mechanisms of cancer genes, particularly within the MYC oncogene family^13^. Indeed, structural controllability has been widely used to analyze many different types of complex biological networks^14–19^.

However, under certain functional requirements, it may be more convenient or practical to concentrate on a specific subset of nodes, known as *target nodes*, for control purposes. Hereafter, we adopt the Maximum Matching (MM) approach to address the problem of target controllability. Target structural controllability introduced by Gao et al. focuses on determining the minimum number of driver nodes necessary to steer the states of the target nodes to their desired states within a finite timeframe^20^, building upon the foundation of structural controllability and utilizing the maximum matching framework as the primary controllability method^1^, which was used in subsequent related research^21,22^.

We define a directed graph $G\left( {V,E} \right)$, where $V$ is the node set, and $E$ is the edge set. Each node $v_{i} { }$ in $V$ is assigned a state value $x_{i} \left( t \right)$ that can depend on time. Therefore, the state vector ${\mathbf{x}}\left( t \right) = [x_{1} \left( t \right)$,…,$x_{N} \left( t \right)] ^{T}$ represents the states of $V = \{ v_{1}$,…,$v_{N} \} .$ Then, the linear system (*A, B, C*) is described by the equations:

1 $$\begin{gathered} \frac{{d{\mathbf{x}}\left( t \right)}}{dt} = A{\mathbf{x}}\left( t \right) + B{\mathbf{u}}\left( t \right) \hfill \\ {\mathbf{y}}\left( t \right) = C{\mathbf{x}}\left( t \right) \hfill \\ \end{gathered}$$

The upper equation indicates that, by considering ${\mathbf{x}}_{0}$ and ${\mathbf{x}}_{{\text{F}}}$ as initial and final states, respectively, and ${\mathbf{u}}\left( t \right) = \left[ {u_{1} \left( t \right), \ldots ,u_{M} \left( t \right)} \right] ^{T}$ as a time-dependent external control input vector of *M* external signals, the system is driven from ${\mathbf{x}}_{0} {\text{to }}{\mathbf{x}}_{{\text{F}}} {\text{in finite time}}.$ Here, A is an *N* × *N* adjacency matrix of the network, and the coupling strength between nodes and external control nodes is denoted by the real matrix *B* with *N* × *M* dimensions. To address target control, the lower equation **y**(*t*) is added into the system (see Eq. 1). In this context, the triplet (*A, B, C*) is referred to as being *target controllable* concerning a designated target node set *S,* where |S| is the cardinality of *S* such that $\left| S \right| \le N$, representing a subset of the set of internal nodes *V* $\left( {S{ } \subseteq { }V} \right)$ (corresponding to the state vector **x**)^20–22^. Target controllability means that there exists a time-dependent input vector **u**(*t*) capable of driving the state of the *target nodes* to any desired final state within a finite timeframe. Therefore, ${\mathbf{y}}\left( t \right) = \left[ {y_{1} \left( t \right), \ldots ,y_{\left| S \right|} \left( t \right)} \right] ^{T}$ denotes the output vector of a target set $S$, and ${\text{C}}$ is an output matrix with $\left| S \right|$ × *N* dimensions. Furthermore, Gao et al. introduced an algorithm designed to identify the smallest driver node set, corresponding to the input vector **u**, within the framework of structural controllability. It is important to note that their algorithm is heuristic in nature and draws from *k*-walk theory, which means it may not always produce optimal solutions^20^. In fact, Czeizler et al. demonstrated that this problem is NP-hard in general^21^, which validates the pursuit of heuristic algorithms in addressing it. On the other hand, Li et al. approached the target control problem by formulating it as the path cover problem and devised a polynomial-time algorithm rooted in network flow analysis^22^. In this context, within a directed graph *G(V, E)* and a set of target nodes *S ⊆ V*, a path cover is defined as a set of disjoint paths and cycles that collectively encompass all nodes within *S*. The objective of the path cover problem is to identify a path cover with the minimal number of paths. While this definition does not precisely mirror the original target control problem, it does establish a sufficient condition for addressing the original problem^22^. In the discussion that follows, we use *P* to represent the set of paths and *Q* to denote the set of cycles within a path cover. Since Refs. ^20–22^ formulate the target control problem in distinct ways, especially Czeizler et al. assume reachability in dealing with cycles while Li et al. do not, there is no contradiction between the NP-hard nature of the original problem and the polynomial-time algorithm devised by Li et al.^22^. Nevertheless, it is worth noting that the algorithm outlined in Ref. ^16^ is intricate in nature. Consequently, we have devised a much simpler algorithm for the path cover problem, which is based on the concept of maximum matching. Subsequently, we have applied this algorithm to several biological networks, with the aim of targeting and controlling specific sets of nodes associated with important biological functions.

Before explaining our computational results and methodology, it is essential to provide a more detailed overview of the existing methodologies. As highlighted earlier, two seemingly contradictory results have been put forth. On one hand, Czeizler et al. asserted that the problem of target structural controllability is NP-hard in general^21^. On the other hand, Li et al. demonstrated that this problem can be solved in polynomial time through a reduction to the maximum flow problem^22^. Notably, there is no inherent inconsistency between these findings as discussed above. This scenario mirrors the distinction in the technical treatment of *driven nodes* versus *driver nodes* discussed in a previous work^23^. Furthermore, there are slight differences between Gao et al. and Czeizler et al. While Czeizler assumes the concept of reachability, Gao’s approach does not rely on it.

In this study, we derive a theorem that enable us to mathematically characterize the problem at hand and, importantly, to demonstrate the correctness of the proposed algorithm, which can resolve the target structural controllability problem efficiently in polynomial time. Furthermore, our algorithm offers a notably simpler solution compared to the one outlined in Li et al.’s work^21^.

In addition to the development of a new algorithm for computing target controllability, this study centers on the application of target controllability methodology to analyze complex real-world biological networks. To put the SimpleTarget algorithm into practical use within real systems, we have selected various networks in which a specific set of nodes can be designated as a target control set. Our selection includes the comprehensive connectome of the nematode worm *C. elegans,* the recently assembled connectome of the *Drosophila* larval brain, and 70 genome-wide metabolic networks spanning significant plant lineages, which were constructed from the Plant Metabolic Network database. Our findings bring to light notable distinctions between the neural systems associated with nematode worms and larval brain insects in terms of the quantity of nodes necessary to control specific functional systems. Furthermore, our analysis uncovers evolutionary trends within the plant lineages, particularly when examining the proportion of nodes required to regulate functional pathways.

## Results

### Theoretical results

In this work, we propose a novel algorithm for target controllability. The SimpleTarget algorithm introduces several innovations compared to that of the standard maximum matching-based algorithm^1^, particularly tailored for achieving target controllability in networks. One distinctive feature is the inclusion of self-loops: SimpleTarget adds self-loops to all non-target nodes during a preprocessing step. Unlike the maximum matching-based algorithm^1^ that focus on controlling entire networks, SimpleTarget specifically identifies the minimum number of driver nodes required for target controllability. This approach not only relies on a simple implementation but also operates efficiently in polynomial time, making it a practical solution for target controllability.

In this section, we summarize our theoretical findings, comprising a mathematical theorem (see Methods section for details). This theorem furnishes a rigorous mathematical proof for the correctness and computational complexity of the proposed algorithm. Our algorithm was developed based on the concept of the path cover problem. Before presenting the path cover problem definition, we will introduce the necessary mathematical notation.

Let *G (V, E)* be a directed graph where *V* is a set of nodes and *E* is a set of directed edges. A sequence of nodes $\left( {v_{{i_{1} }} ,v_{{i_{2} }} , \ldots ,v_{{i_{k} }} } \right)$ is called a path if $v_{{i_{p} }} \ne v_{{i_{q} }}$ holds for all $p \ne q$ and $\left( {v_{{i_{p} }} , v_{{i_{p + 1} }} } \right)$ ∈ *E* holds for all $p = 1, \ldots , k - 1.$ Similarly, a sequence of nodes $\left( {v_{{i_{1} }} ,v_{{i_{2} }} , \ldots ,v_{{i_{k} }} , v_{{i_{1} }} } \right)$ is called a cycle if $v_{{i_{p} }} \ne v_{{i_{p + 1} }}$ holds for all $p \ne q, _{ }$ $\left( {v_{{i_{p} }} , v_{{i_{p + 1} }} } \right)$ ∈ *E* holds for all $p = 1, . .., k - 1_{ }$, and $\left( {v_{{i_{k} }} , v_{{i_{k + 1} }} } \right)$ ∈ *E* holds. Then, the path cover problem is defined as below^22^.

#### Definition 1 (*Path Cover Problem*)

Given a directed graph *G(V, E)* and a set of target node *S ⊆ V* , find a set of disjoint cycles and paths with the minimum number of paths that include all nodes in *S.*

**Figure 1:**
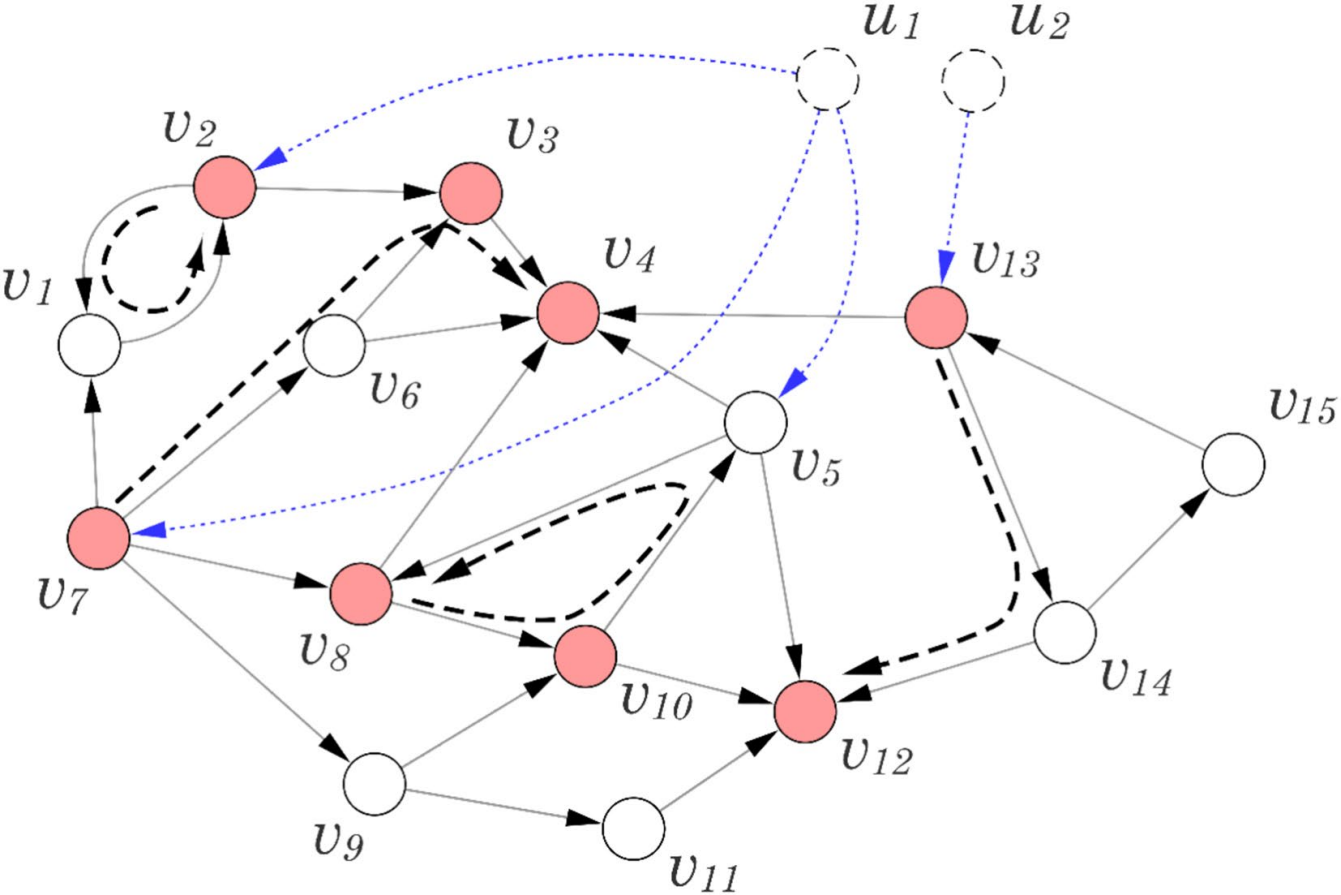
Example of the path cover problem. In this figure, filled (red) nodes represent target nodes and dotted nodes (i.e., $u_{1}$, and $u_{2}$) represent external control nodes. In this case, target nodes are covered by two cycles and two paths. As in Refs. ^1,20,22^, it is assumed that all cycles and one path are controlled by one external node. Therefore, we only need two external control nodes.

Note that initial nodes in distinct paths should be controlled by distinct external nodes whereas all cycles can be controlled by one external node, as assumed in Refs. ^1,20,22^. An example of the path cover problem is shown in Fig. 1. The network consists of eight target nodes that can be controlled by two external control nodes $u_{1}$ and $u_{2} .$ In this particular example, the target nodes are covered by a combination of two cycles and two paths. Following the methodology described in Ref.^1^, we assume that all cycles and one path can be controlled by a single external node. Consequently, in this example we only require two external control nodes $u_{1}$ and $u_{2} .$

By utilizing the aforementioned path cover concept, we have devised a novel algorithm named SimpleTarget, which offers a simpler and efficient solution to the target structural controllability problem. The full details of the SimpleTarget algorithm are outlined in the Methods section. Then, by combining this path cover concept with the maximum matching-based controllability approach^1^, we establish the following theorem:

#### Theorem 1

*Algorithm* *SimpleTarget solves the target structural controllability problem (in the sense of the path cover problem shown in  Ref.*^22^*) in polynomial time.*

A detailed proof of the theorem can be found in the Methods section.

We have computed the simulation results (state evolution trajectories) to show that the network can indeed be target controlled. This demonstrates that the nodes identified by the proposed SimpleTarget algorithm are the ones that drive the target node states to the desired final state. The detailed analysis is provided in the Supplementary Information file (Supplementary Fig. S1).

### Computational results derived from data analysis

#### Metrics to evaluate target control

Gao et al. primarily focused their analysis of target controllability on artificially generated networks, rather than on experimental biological networks^20^. Consequently, they adopted two distinct methodologies for selecting a set of target nodes. In one approach, they *randomly* selected a fraction *f* of nodes. In a different scenario, they implemented a so-called *local schema*, which still involved random selection of a fraction *f* of nodes, but with the additional condition that these nodes must be adjacent. Here *f* is defined as* f* = *N*_*T*_/*N* where *N*_*T*_ is the number of target nodes and *N* the total number of nodes. To gauge the efficiency of target control for a specific fraction *f*, they introduced the target controllability parameter, denoted as $\alpha_{D}$. This parameter is defined as $\alpha_{D}$ = *P*_*D*_*/N*_*D*_, where* P*_*D*_ represents the minimum number of driver nodes required to control a fraction *f* of target nodes, while *N*_*D*_ represents the minimum number of driver nodes needed to control the entire network^20^. As such, $\alpha_{D}$ serves as a measure of how efficiently target control operates in comparison to full control over the entire network. It quantifies the ratio of driver nodes needed for targeted control to those needed for controlling the entire network. This parameter was employed to evaluate both random and local control schemas in the analyzed scale-free (SF) and Erdős-Rényi (ER) networks ^20^. In our data analysis, we adopt $\alpha_{D}$ to assess target control in biological networks. Additionally, we introduce $\beta_{D}$, defined as $\beta_{D}$ = $\alpha_{D}$/*f*, to assess how target control efficiency compares to a neutral expectation. When $\beta_{D}$ is less than 1, it signifies that target control is more efficient than neutral expectation. If target control operates as efficiently as the neutral expectation ($\beta_{D}$ = 1), it implies that

$$P_{D} = fN_{D} ,\,{\text{and}}\,\alpha_{D} = f.$$

In our research, our objective is to apply the target controllability algorithm to real biological networks. Specifically, we analyze neural networks from the *C. elegans* worm^12,24,25^ and the *Drosophila* fly insect^26^, as well as metabolic networks associated with 70 different plant organisms (further details are available in the Methods section)^27,28^. To achieve this, we must carefully select sets of target nodes related to specific biological functions or pathways of interest.

### Results for the analysis of connectome networks

The initial segment of our analysis focuses on connectome networks. The neuronal network of the *C. elegans* worm is composed of 378 nodes and 5256 directed links, and consists of various functional neuron classes, including motor, interneuron, poly-modal, sensory, and muscle neurons^12,24,25^. As part of our analysis, we designate each of these classes as a set of target nodes for control purposes. Another dataset is derived from the recently discovered connectome of the larval brain of the Drosophila melanogaster insect, comprising a comprehensive network of 2952 neurons and 110,140 directed links^26^. Similar to the approach taken with the *C. elegans* worm, we establish specific sets of target nodes based on the functional neuron classification in this network. These classes are further categorized into three major groups: brain inputs, interneurons, and brain outputs. From a target control perspective, it is intuitive to consider the brain outputs as potentially more desirable for control.

**Figure 2:**
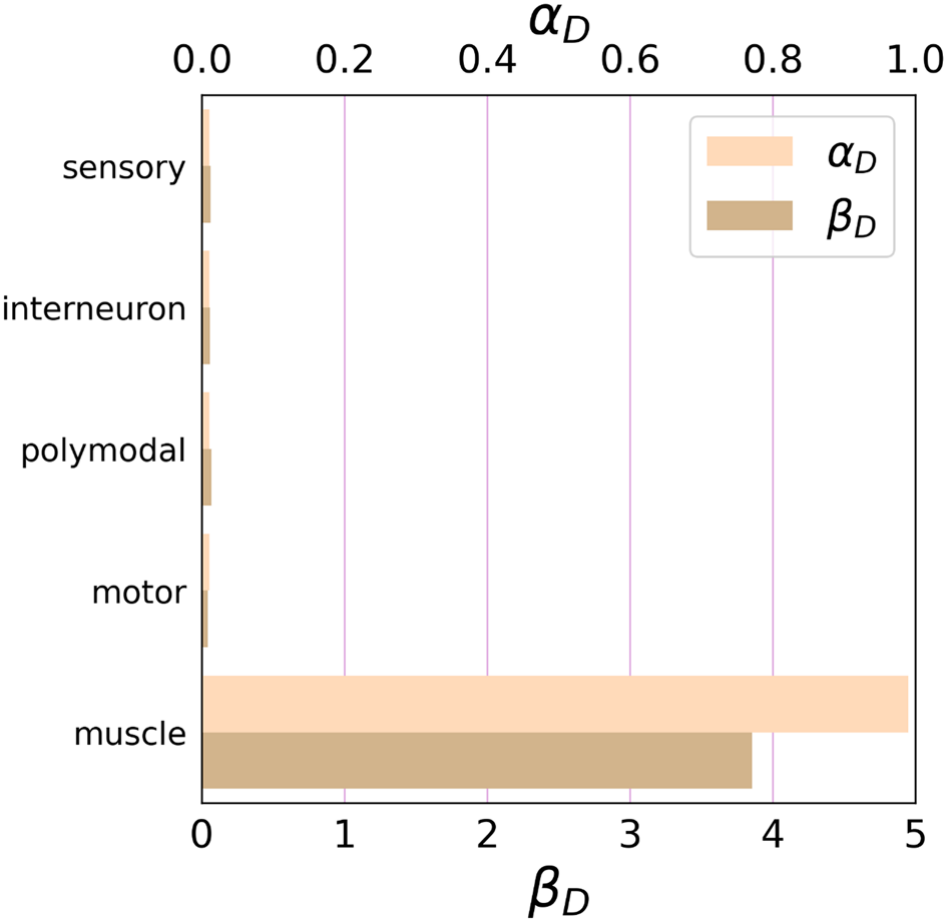
Computed $\alpha_{D}$ and $\beta_{D}$ metrics for each functional neuron class considered as controllability target set. Notably, muscle neurons display a discernible trend of being accessible to targeting by a set of driver nodes, which is plausible for real-world applications.

**Figure 3:**
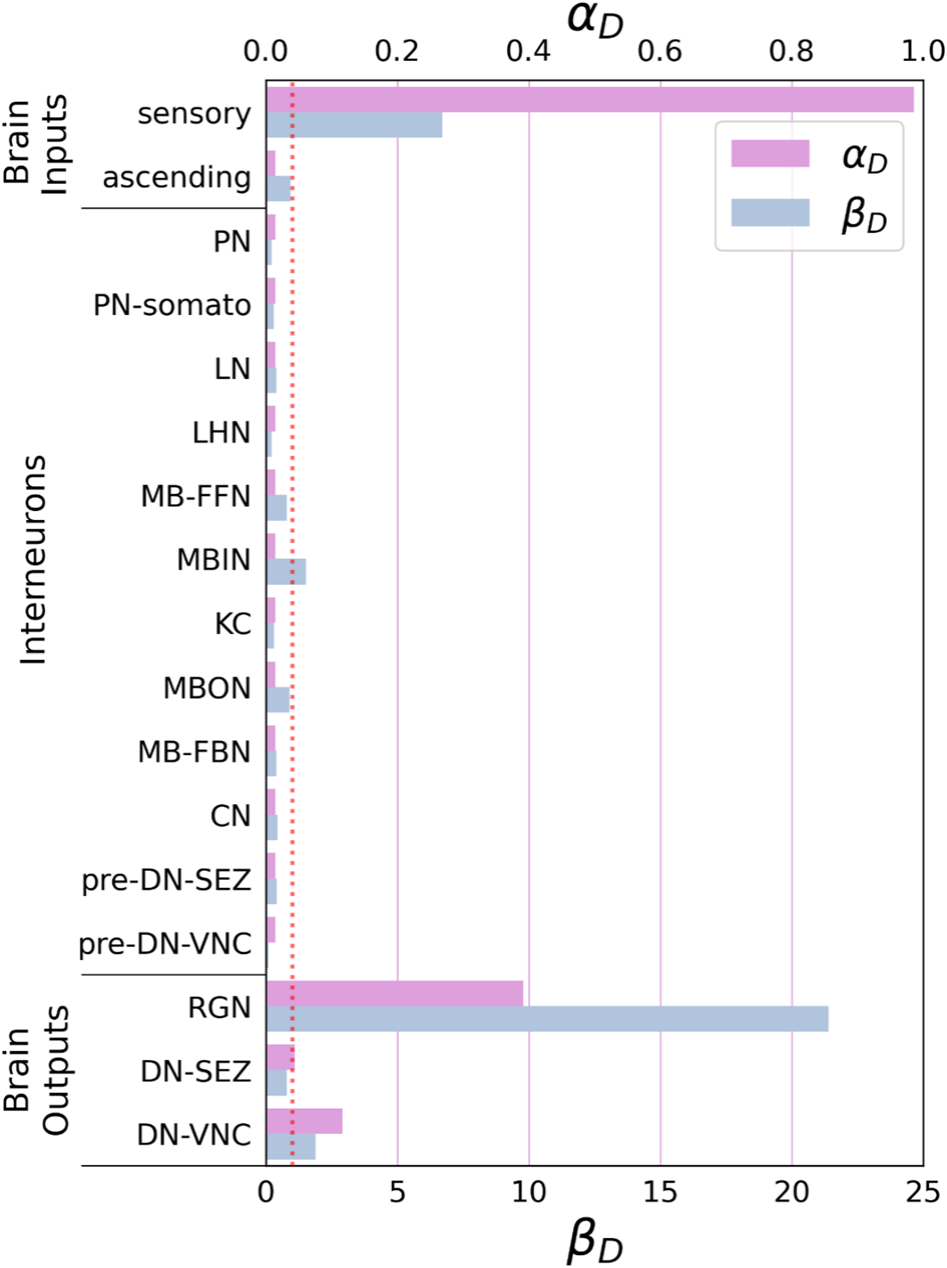
Computed $\alpha_{D}$ and $\beta_{D}$ metrics for each functional neuron class considered as controllability target set in the brain of the fruit fly insect (*Drosophila*). All classes can be categorized into three main groups: input neurons, interneurons, and output neurons. Many classes exhibit controllability by a single external control node (*P*_*D*_ = 1) or few nodes, reflecting the existence of loops in the internal network structure. Notably, all three classes belonging to the output neuron group (RGN, DN-SEZ, DV-VNC) can be target-controlled by a larger set of driver nodes. The dashed red line represents $\beta_{D}$ = 1.

**Figure 4:**
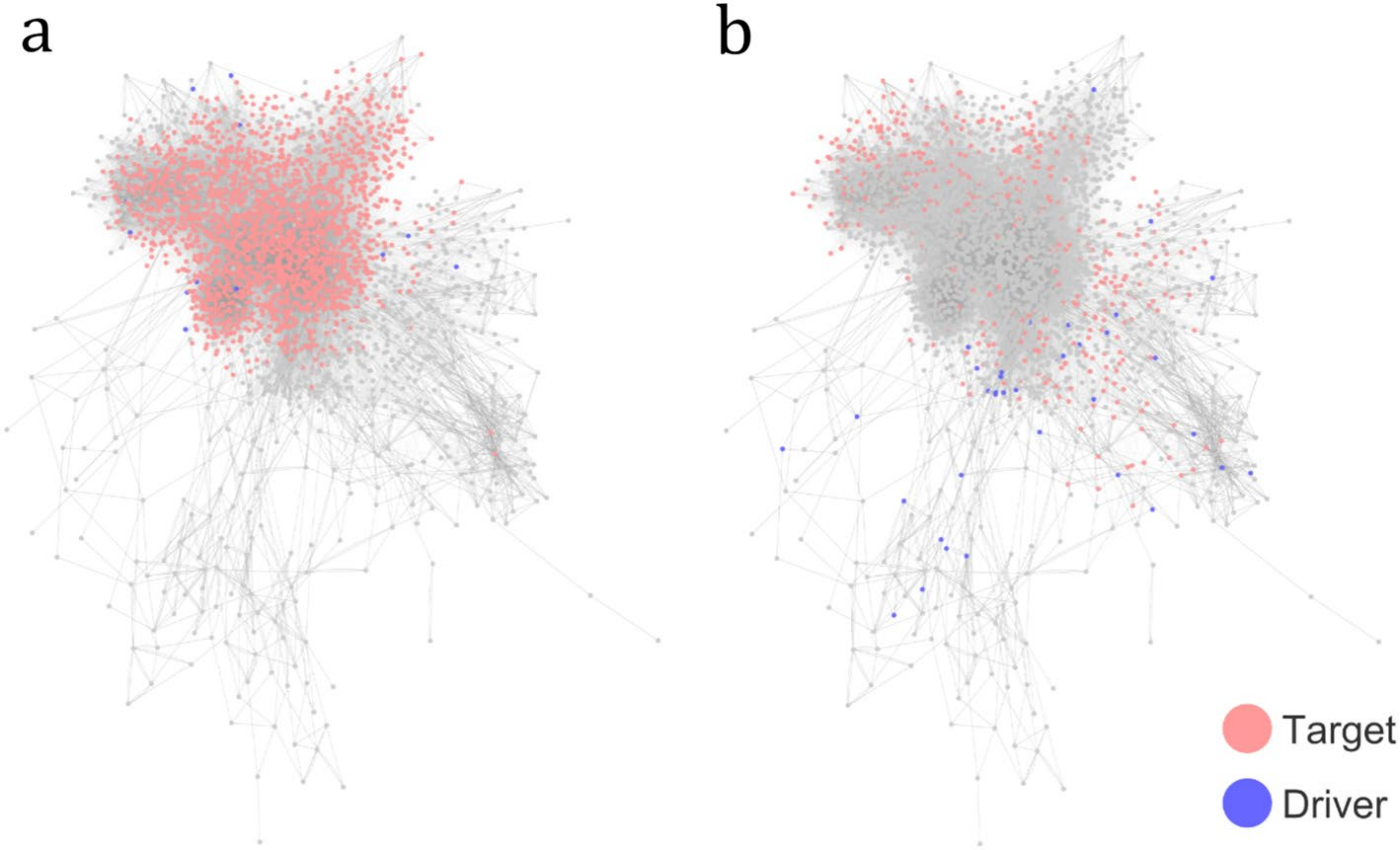
Visualization of the entire *Drosophila* connectome with highlighted target control sets (red) and the driver node set (blue). (**a**) The target control set of interneurons. (**b**) The target control set of brain output neurons. Notably, the brain output neurons are mapped into more distributed locations and require more driver nodes. In contrast, interneurons are more interconnected, which favors the presence of loops.

In the case of *C. elegans*, when we consider muscles as the target control set, the SimpleTarget algorithm yields a result of $\alpha_{D}$ = 0.989 (as shown in Fig. 2). Shifting our focus to the *Drosophila* brain network, we select brain output neuron classes as the target control sets, resulting in the following outcomes: Ring Gland Neurons (RGN) yield $\alpha_{D}$ = 0.391, Descending Neurons to Subesophageal Zone (DN-SEZ) result in $\alpha_{D}$ = 0.043, Descending Neurons to Ventral Nerve Cord (DN-VNC) yield $\alpha_{D}$ = 0.115 (see Fig. 3). These results suggest that the *Drosophila* connectome appears to be more optimized for brain output neuron control when compared to the muscle class for *C. elegans*. When assessed in the context of the neutral expectation, DN-SEZ gives a $\beta_{D}$ = 0.782, indicating a level of control efficiency below one. In the case of *C. elegans*, the result for muscles a as target control is $\beta_{D}$ = 3.857, quite above the neutral efficiency threshold of 1. On the other hand, DN-VNC and RGN neuronal classes generate values exceeding one, with $\beta_{D}$ = 1.88 and 21.39, respectively. While a precise quantitative comparison between the connectomes of both organisms proves challenging due to their intrinsic biological differences, a qualitative assessment suggests that *C. elegans* demonstrates superior efficiency in controlling input neurons related to sensory classes compared to *Drosophila* (see brain inputs and sensory classes in Figs. 2 and 3). Intuitively, it seems reasonable that the $\alpha_{D}$ is for sensory neurons in *Drosophila* is large since these neurons primary receive stimuli from the external environment and may not have internal input edges. However, in *C. elegans* the observation of a small value of $\alpha_{D}$ can be attributed to an information transfer between sensory-sensory connections, which develop a higher abundance of loops or between sensory nodes and other neurons. The prevalence of loops in this particular class may contribute to the enhanced efficiency of input neuron control observed in *C. elegans* as compared to *Drosophila*. In contrast, optimization in interneuron control is observed in both *Drosophila* and *C. elegans*, requiring minimal external control nodes (see Fig. 4), which is originated by the existence of loops. Furthermore, as discussed above, when evaluating output neurons, the *Drosophila* connectome appears to exhibit greater efficiency than its *C. elegans* counterpart.

**Figure 5:**
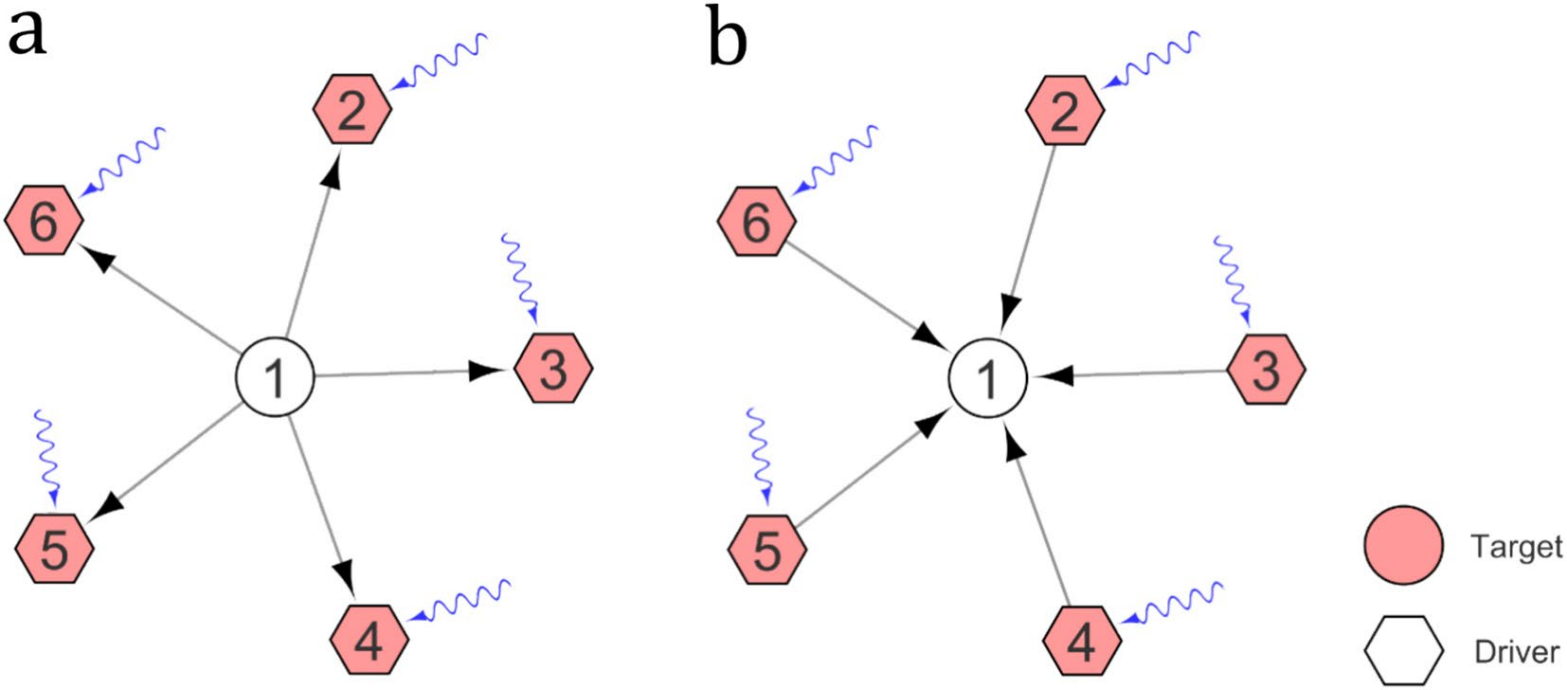
In the example, both (**a**) and (**b**) have P_*D*_ = 5, *N*_*D*_ = 5, so $\alpha_{D} =$ 1. However, (**a**) has output nodes as the target, while (**b**) has input nodes as the target.

The elevated $\alpha_{D}$ observed in sensory nodes of *Drosophila* is due to their limited number of input edges. Conversely, in *C. elegans*, the high observed in muscles $\alpha_{D}$ is a consequence of their sparse output edges. Although the result, a large $\alpha_{D}$, is the same, the underlying mechanisms differ between the two scenarios. This nuanced contrast, where a high $\alpha_{D}$ may arise from either a scarcity of input edges or a shortage of output edges (see Fig. 5), adds an interesting dimension to the comparative analysis.

Notably, it is intriguing to observe that several target node sets linked to functional categories, such as interneurons, within *C. elegans* and Drosophila exhibit *P*_*D*_ = 1. This observation suggests that these target neuronal sets themselves inherently possess controllability. That is, these subsets of neurons contain loops and, therefore, can be controlled by a single external node.

To further investigate the topological features of the identified driver nodes, we compared the mean degree of driver nodes < $k_{D}$ > to the mean degree of each functional neuron class (i.e., target node set) < $k_{T}$ > in the *C. elegans* neuronal network and the *Drosophila* brain connectome. The results shown in Fig. S2 (Supplementary Information) indicate that relatively low-degree nodes are used to control specific target systems. In other works, the driver nodes tend to avoid hubs.

### Results for the analysis of metabolic networks from 70 plant species

**Figure 6:**
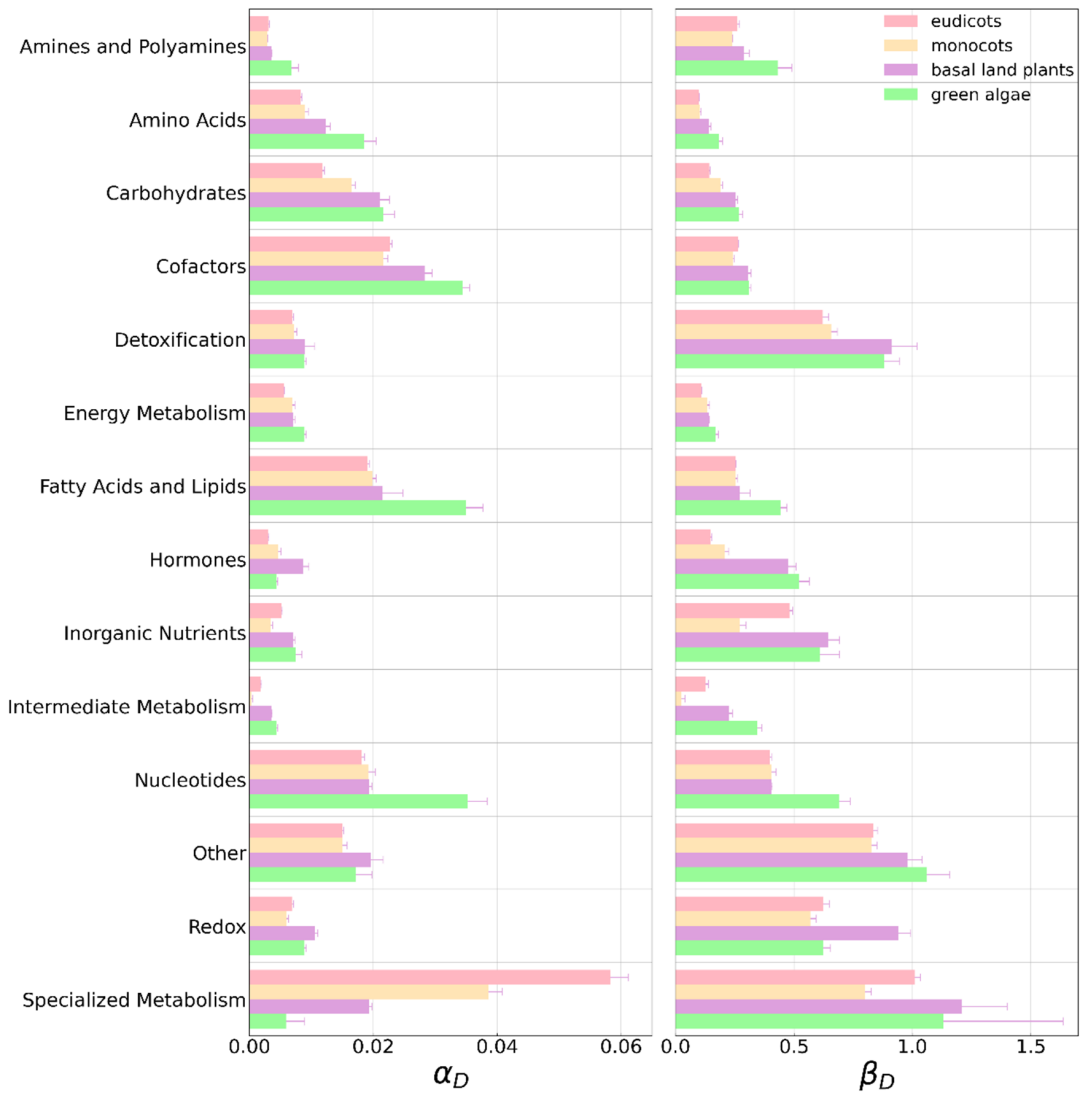
Computed $\alpha_{D}$ and $\beta_{D}$ for plant metabolic networks. The target sets are specific functional pathways and the enzymes/reactions involved in them. Colors denote the four major lineages. It seems there is an evolutionary tendency because $\alpha_{D}$ and $\beta_{D}$ metrics tend to increase in most pathways from eudicots/monocots (most modern) plants to basal plants and green algae (more primitive).

By using plants metabolic networks, we may also set as target nodes specific pathways or enzyme classes^27,28^. Figure 6 displays the $\alpha_{D}$ and $\beta_{D}$ metrics computed for plant metabolic networks. The target sets represent specific functional pathways and the enzymes/reactions associated with them. An intriguing trend is observed, suggesting an evolutionary tendency. The $\alpha_{D}$ and $\beta_{D}$ metrics appear to increase as we move from the pathways of eudicots/monocots (more modern) to those of basal plants and green algae (more primitive). Moreover, overall, the values of $\alpha_{D}$ are much smaller compared to those of connectome networks, suggesting an abundance of loops.

**Figure 7:**
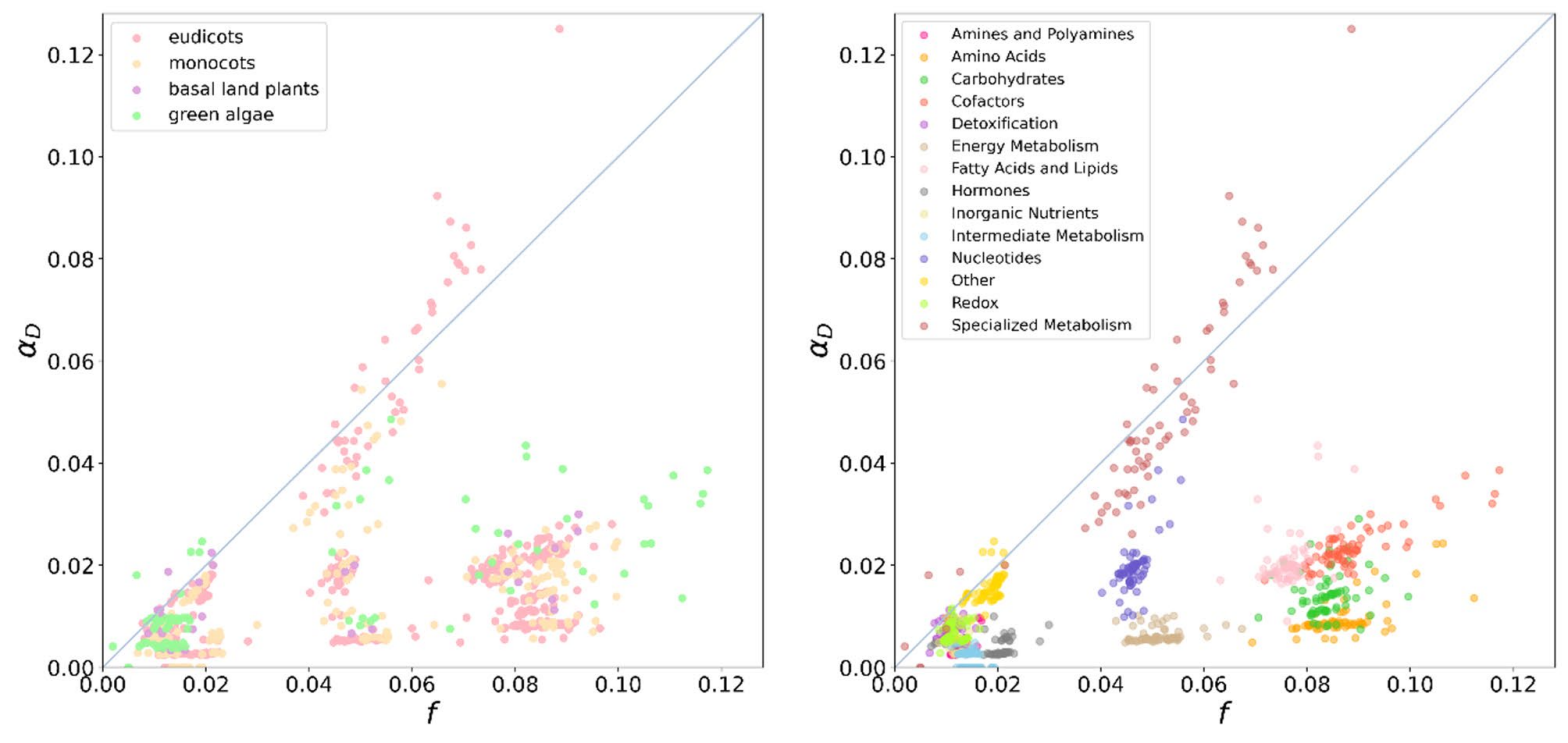
Scattered plot between $\alpha_{D}$ metric and *f* computed for plant metabolic networks. (Left) Each dot corresponds to a pathway and there are 70 plants so we have 70 × 14 data points. They are colored by main lineage types. (Right) The dots are colored according to the pathway functional class indicated in legend.

On the other hand, Fig. 7 shows a scatter plot that illustrates the relationship between the $\alpha_{D}$ metric and *f* calculated for the full set of plant metabolic networks. In the left panel, each dot corresponds to a pathway, resulting in a total of 70 × 14 data points, with colors representing the main lineage types. The right panel, on the other hand, uses colors to differentiate the data points based on the functional class of the pathways. While it may not be apparent that plants from major lineage groups follow a distinct pattern (Fig. 7(left), the scatter plot that includes indications for functional pathways reveals a more clustered trend (Fig. 7(right)). In other words, pathways with the same biological functions tend to exhibit similar $\alpha_{D}$ versus *f* values. Moreover, most of them tend to be more efficient than the neutral expectation $\left( {\alpha_{D} < f} \right)$, with some samples of specialized metabolism being exceptions.

To get deeper insights into the network features of the identify driver nodes, we compared the mean degree of driver nodes < $k_{D}$ > to the mean degree of functional pathways (i.e., target node sets) < $k_{T}$ > in the analyzed plant metabolic networks. Similar to the results observed in neural networks (Fig. S2), the findings shown in Fig. S3 (Supplementary Information) suggest that high-degree nodes are not commonly selected as driver nodes for controlling specific plant metabolic pathways. This observation becomes clearer when comparing the degree distributions of driver nodes with those of the target nodes associated with energy metabolism. As shown in Fig. S4 (Supplementary Information file), the degree distribution of plant metabolic networks across main lineages follows a power-law distribution. This implies that a small fraction of nodes are highly connected (i.e., hubs). Interestingly, our results show that these nodes are typically not chosen as driver nodes (see Figs. S5–S7 in the Supplementary Information). Instead, most sets of driver nodes are small in size and tend to exhibit low or medium-degree values.

## Discussion and conclusion

In this work, we proposed a simple yet efficient algorithm for target control based on the path cover problem. The algorithm was applied to conduct an extensive data analysis of real-world biological networks, focusing on specific sets of functional nodes as target control. Notably, our study used the recently available comprehensive data on the entire neuronal brain of the *Drosophila* insect, offering a unique comparison with other neuronal systems such as *C. elegans* worm, along with plants metabolic networks from various major lineages.

In conclusion, the observed large $\alpha_{D}$ for sensory neurons in *Drosophila* aligns with their primary role in receiving stimuli from the external environment, potentially indicating a lack of internal input edges. Conversely, the small $\alpha_{D}$ in *C. elegans* suggests efficient information transfer within sensory-sensory connections, fostering a higher abundance of loops and interactions with other neurons. This prevalence of loops likely contributes to the superior efficiency of input neuron control in *C. elegans* compared to that in *Drosophila*. While both organisms demonstrate optimized interneuron control, requiring minimal external control nodes due to the presence of loops, *Drosophila*’s connectome exhibits greater efficiency in output neurons than its *C. elegans* counterpart, highlighting varied control strategies in different neuronal classes.

In the analysis of metabolic pathways, an evolutionary trend is evident as both $\alpha_{D}$ and $\beta_{D }$ metrics consistently decrease across most pathways. This trend is observed from more primitive organisms, such as basal plants and green algae, towards the more modern eudicots/monocots plants, with specialized metabolism possibly being an exception. The decrease in $\alpha_{D}$ suggests an enhancement in target control efficiency across evolution. Furthermore, across lineages, almost all pathways exhibit $\beta_{D}$ values smaller than 1, indicating that target control for each pathway is more efficient than the neutral expectation. Notably, this efficiency appears to have been enhanced throughout evolution.

As mentioned above, some specialized metabolic pathways shown an exception of the observed trend. Specialized metabolic pathways, also known as secondary metabolism, exhibit greater diversity compared to primary metabolic pathways for several reasons. They generate structurally varied compounds linked to specific ecological roles such as defense, signaling, and adaptation to environmental stresses, resulting in higher chemical complexity^29,30^. Unlike the primary metabolism, secondary metabolism shows increased complexity, with pathways forming distinct modules that can evolve independently, leading to significant diversity^31^. Furthermore, enzymes involved in specialized metabolism often display high substrate specificity, and gene duplication followed by divergence enhances the evolution and complexity of these pathways^32^. These significant differences between secondary metabolism and primary metabolism may have reshaped the topology of these pathways, influencing their controllability in distinct ways, as observed in our study.

In summary, by combining a novel and efficient algorithm to tackle target control problem with data analysis from connectomes and metabolic pathways in plants, our data-driven study represents a novel effort in providing insights into control features and strategies for achieving precise target control of specific functional segments in complex neuronal networks and metabolic pathways. The approach, with its easy implementation and fast computation, holds promise for future applications in diverse biological networks and genome-scale pathways.

## Methods

### Datasets

The neural network in the *C. elegans* worm comprises 378 nodes and 5256 directed links, encompassing diverse functional neuron classes such as motor, interneuron, poly-modal, sensory, and muscle neurons and was collected from the WormAtlas database^25^. In our analysis, we categorize each of these classes as a set of target nodes for control objectives. Additionally, we utilize another dataset extracted from the recently unveiled connectome of the larval brain of the *Drosophila melanogaster* insect. This connectome presents a comprehensive network featuring 2952 neurons and 110,140 directed link and was downloaded from supplementary material file of Ref.^26^. Note that multi-links connecting the same pairs of neurons were excluded, and each directed edge in our analysis was assigned a weight of one. The data for plant metabolic networks is publicly available in the PMN database^28^. We constructed enzyme-reaction-centric metabolic networks for 70 plant species, encompassing four major lineages, including 2 algae, 6 basal land plants, 12 monocots, and 50 eudicots.

### SimpleTarget algorithm

Our proposed algorithm is quite simple. The algorithm is called SimpleTarget and its pseudocode is given as below.

Step 1: Add self-loops to all non-target nodes.

Step 2: Determine the minimum number of driver nodes using the maximum matching-based algorithm in^1^.

**Figure 8:**
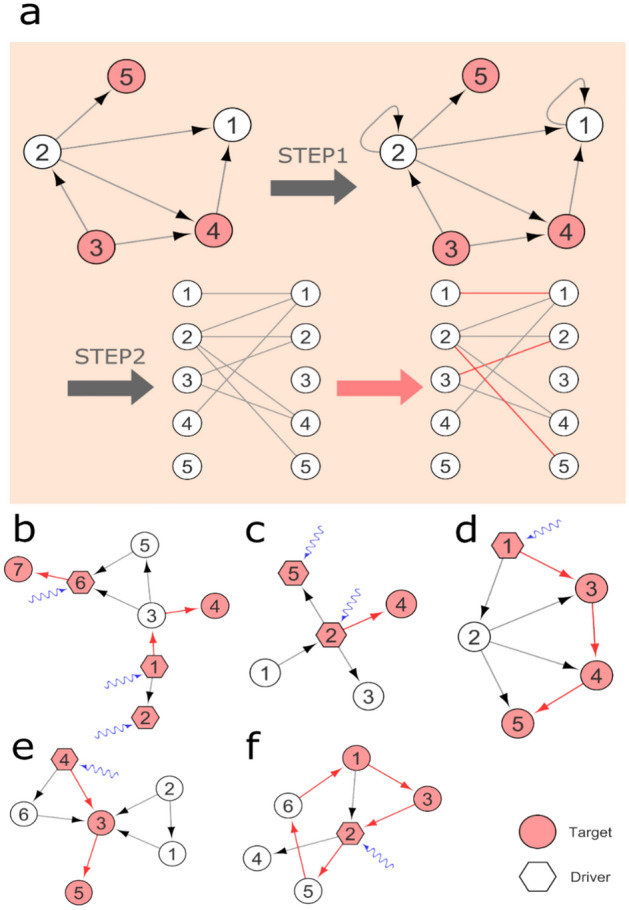
(**a**) Illustration of the proposed algorithm for target controllability. Step 1 adds self-loops to all non-target nodes. Step 2 determines the minimum driver nodes based on maximum matching-based algorithm^1^. (**b**–**e**) Several examples of directed networks wherein our algorithm identifies a solution. (**f**) An illustrative graph instance wherein the count of driver nodes *P*_*D*_ is one. Interestingly, we observed that several target node sets associated with functional categories of *C. elegans* and *Drosophila* exhibit *PD* = 1 (see Figs. 2 and 3).

This algorithm works in polynomial time. The algorithm is illustrated in Fig. 8a, and some graph examples are also shown in Fig. 8b–f.

For the sake of self-completeness, we briefly explain the maximum matching-based algorithm in Ref.^1^. We construct a bipartite graph *BG*$\left( {V_{L} , V_{R} , E_{b} } \right)$ from *G*(*V, E*) by $V_{L}$ = $\left\{ {v_{i}^{L} |v_{i} \in V} \right\}$, $V_{R}$ = $\left\{ {v_{i}^{R} |v_{i} \in V} \right\}$, $E_{B}$ = $\left\{ {\left( {v_{i}^{L} , \,v_{j}^{R} } \right)|\left( {v_{i} , v_{j} } \right) \in V} \right\}$ (see Fig. 8a). A subset of edges *M* ⊆ $E_{B}$ is called a matching (in a bipartite graph) if any two edges in *M* do not share any endpoint. A maximum matching is a matching with the maximum number of edges. It is well-known that a maximum matching can be computed for a bipartite graph (and also for an undirected graph) in polynomial time^33^. Finally, the unmatched nodes in $V_{R}$ correspond to the driver nodes, which should be controlled by distinct external nodes.

The detailed pseudocode of the algorithm is also given as follows:
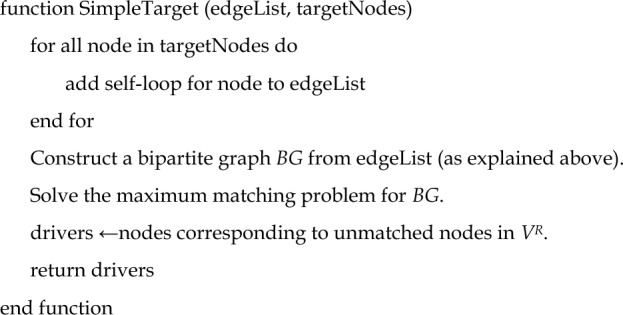


Note that the full code, including the detailed code necessary to solve the maximum matching-based algorithm^1^, is publicly available on GitHub. See the Data Availability section for details.

#### Theorem 1

*Algorithm* SimpleTarget *solves the target structural controllability problem (in the sense of the path cover problem shown in Ref. *^22^*) in polynomial time.*

#### *Proof*

It is straight-forward to observe that the algorithm works in polynomial time (since the algorithm in Ref. ^1^ operates in polynomial time). Therefore, we prove that SimpleTarget consistently finds an optimal path cover (i.e., a path cover with the minimum number of paths). Note that the algorithm in^1^ always finds a path cover with the minimum number of paths for a given directed graph when *S* = *V*.

In this proof, let *G(V, E)* and *S*_*0*_ be the input graph and the target node set in the original path cover problem, respectively. First, let *G(V, E′)* be the directed graph obtained from *G(V, E)* by adding self-loops for all nodes not appearing in S_0_. Let (*P0, Q0*) be an optimal solution found by the algorithm in Ref. ^1^ for *G(V, E′)*. Then, we remove self-loops in *Q*_*0*_ for the nodes not appearing in *S*_*0*_. Let *Q*_*1*_ be the resulting set of cycles. Consequently, it is obvious that (*P*_*0*_,*Q*_*1*_) forms a path cover for *G(V, E)* and *S*_*0*_. This means that SimpleTarget gives a solution (but not necessarily optimal) for the path cover problem.

Next, let *(P, Q)* be an optimal solution for the path cover problem for *G(V, E)* and *S*0. Then, we add self-loops to the nodes not appearing in *P ∪ Q*. Let *G(V, E′′)* be the resulting graph. Given that all target nodes appear in *P ∪ Q*, *E′′ ⊆ E ′* holds. Let *Q′* be the set of added self-loops. Then, (*P, Q ∪ Q′*) is clearly a path cover for *G(V, E′)* with *S* = *V.* As (*P*_*0*_, *Q*_*0*_) is an optimal path cover for *G(V, E′)* with *S* = *V* , *P*_*0*_ ≤*|P|* holds. Therefore, (*P*_0_, *Q*_1_) is an optimal path cover for *G(V, E)* and *S*_*0*_, which means that SimpleTarget provides an optimal solution for the path cover problem. *q.e.d*.

### Supplementary Information

Supplementary Information 1.

Supplementary Information 2.

## Data Availability

The custom code for the developed algorithm used in this study is publicly available from: https://github.com/wataru-s1/SimpleTarget. Our study did not generate new biological dataset; therefore, the manuscript does not report biological data generation. The data used in this study is publicly available from the WormAtlas^25^ and the PMN^28^ databases, and from a previous publication (Supplemental Material section) referenced in the text^26^: 10.1126/science.add9330
