# Supplementary material for: Target control of linear directed networks based on the path cover problem
Source: Sci Rep. 2024 Jul 23;14:16881. doi: 10.1038/s41598-024-67442-7 (PMC11266607; doi:10.1038/s41598-024-67442-7)
Supplement: Supplementary file 1 — Supplementary Information 1. [file 41598_2024_67442_MOESM1_ESM.pdf]

Supplementary Information

Target control of linear directed networks based on the path cover problem

Wataru Someya[1], Tatsuya Akutsu[2] & Jose C. Nacher[1*]
[1]*Department of Information Science, Faculty of Science, Toho University, Funabashi, Chiba 274-8510, Japan.*
[2]*Bioinformatics Center, Institute for Chemical Research, Kyoto University, Kyoto, Uji, 611-0011, Japan. *Corresponding author*

Numerical Example of the Dynamics for Target Controllability.

We show an example of how to target control by driving the state of a specific set of nodes to a desired final state $\mathbf{y}_f$. In general, matrices *A, B,* and *C* are defined as below.

- *A, B, C* are *N* × *N, N* × *M*, and |S| × N matrices, where *N* is the number of internal nodes, *M* is the number of external nodes (control nodes), and *S* is a set of target nodes.
- A_{ij}= 1 if there is a directed edge (v_j, v_i); otherwise A_{ij}= 0.
- B_{im} is non-zero if and only if there is a directed edge (u_m, v_i).
- $C_{ik} = 1$ if $v_k$is the ith target nodes; otherwise $C_{ik} = 0$.

Note that $\mathbf{x}(t)$, and $\mathbf{u}(t)$ are *N*-dimensional, $|S|$-dimensional, and *M*-dimensional vectors, respectively. The relations between these vectors and *A, B,* and *C,* are explained in Eq. (1) of the main text.

Consider the network in Supplementary Fig. S1a. In this case, we regard v_2, v_3, v_7, and v_8 as the first, second, third, and fourth target nodes, respectively. Then, *A, B,* and *C* will be as follows:

$$A = \begin{bmatrix} 0 & 1 & 0 & 0 & 0 & 0 & 0 & 0 \\ 1 & 0 & 0 & 0 & 0 & 0 & 0 & 0 \\ 0 & 1 & 0 & 0 & 1 & 0 & 0 & 0 \\ 0 & 0 & 0 & 0 & 1 & 0 & 0 & 0 \\ 1 & 0 & 0 & 0 & 0 & 1 & 1 & 0 \\ 0 & 0 & 0 & 0 & 0 & 0 & 1 & 0 \\ 0 & 0 & 0 & 0 & 0 & 0 & 0 & 0 \\ 0 & 0 & 0 & 1 & 0 & 1 & 0 & 0 \end{bmatrix}$$

$$B = \begin{bmatrix} B_{11} & 0 \\ 0 & 0 \\ 0 & 0 \\ 0 & B_{42} \\ 0 & 0 \\ 0 & 0 \\ B_{71} & 0 \\ 0 & 0 \end{bmatrix}$$

$$C = \begin{bmatrix} 0 & 1 & 0 & 0 & 0 & 0 & 0 & 0 \\ 0 & 0 & 1 & 0 & 0 & 0 & 0 & 0 \\ 0 & 0 & 0 & 0 & 0 & 0 & 1 & 0 \\ 0 & 0 & 0 & 0 & 0 & 0 & 0 & 1 \end{bmatrix}$$

In this case, $\mathbf{x}(t)$, $\mathbf{y}(t)$, and $\mathbf{u}(t)$ are 8-dimensional, 4-dimensional, and 2-dimensional vectors, respectively. In this example, we set $\mathbf{x}(0)=\mathbf{0}$ and the final desired state $\mathbf{y}(\mathrm{t}_f) \equiv \mathbf{y}_f$ as the vector (1,1,1,1). It is well-known that by using the following $\mathbf{u}(t)$ signal [20]:

$$\boldsymbol{u}(t) = B^T e^{A^T(t_f - t)} C^T (CWC^T)^{-1} \boldsymbol{y}_f \qquad \text{Eq. S1}$$

where $W(s)= \int_0^s e^{A\rho} B B^T e^{A^T \rho} d\rho$ is the controllability *Gramian* matrix, we can numerically solve Eq. 1. Fig. S1b shows that the state vector of the target nodes reaches the desired final state at time t_f=10 by using two external signals. Note that the target nodes can be covered by a combination of one cycle and two paths, which, according our proposed algorithm, can be controlled by two external control nodes, u_1 and u_2. Note that in the computational experiment we used non-zero random values for the elements of matrices A_{ij} and B_{ij}.

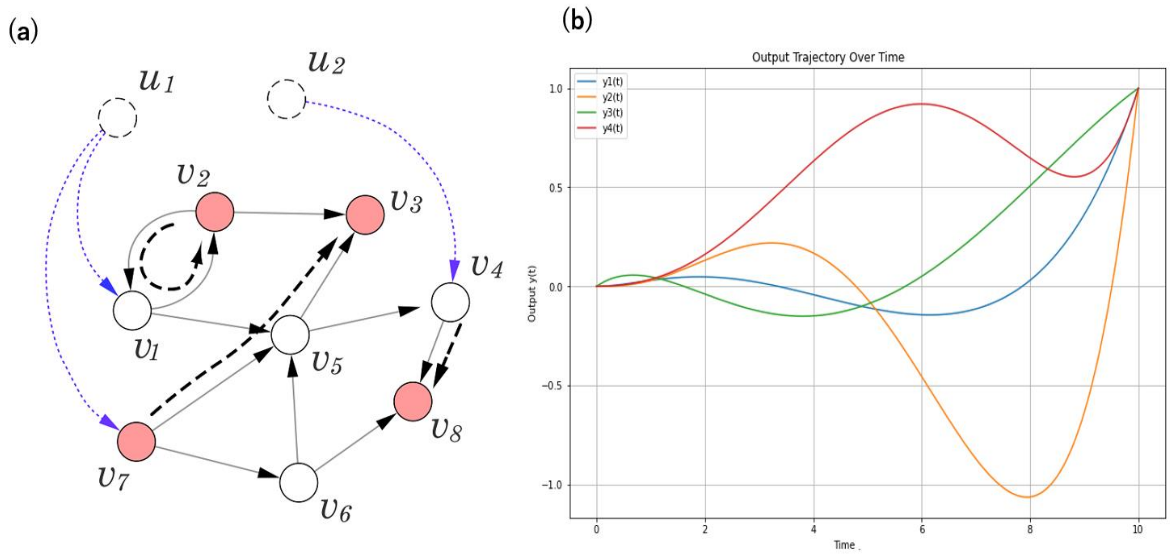

Supplementary Figure S1. (a): Example of a graph showing the relationship between (A, B, C) and the path cover problem. The meanings of types of nodes and edges are the same as those in Fig. 1 of the main text. The network consists of four target nodes that can be controlled by two external control nodes u_1 and u_2. In this particular example, the target nodes are covered by a combination of one cycle and two paths. (**b**). Numerical results of Eq. 1 in main text using Eq. S1 as the external signal. The state vector of the four target nodes reaches the desired final state at time t_f=10.

Additional network analysis based on node degree

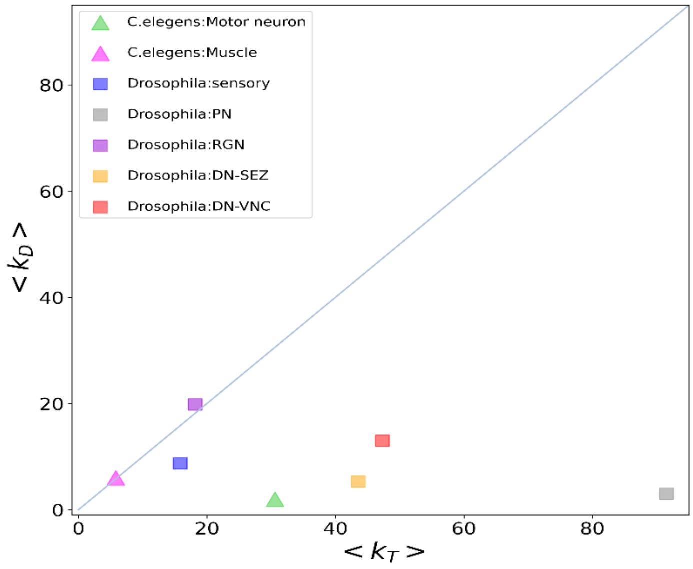

Supplementary Figure S2. The mean degree of driver nodes $<k_D>$ is compared to the mean degree of each functional neuron class (i.e., target node set) $<k_T>$ in the *C. elegans* neuronal network (triangles) and the *Drosophila* brain connectome (squares). The results indicate that relatively low degree nodes are used to control specific target systems. In other works, the hubs are avoided by the driver nodes. Functional classes controlled by a single external node are not shown.

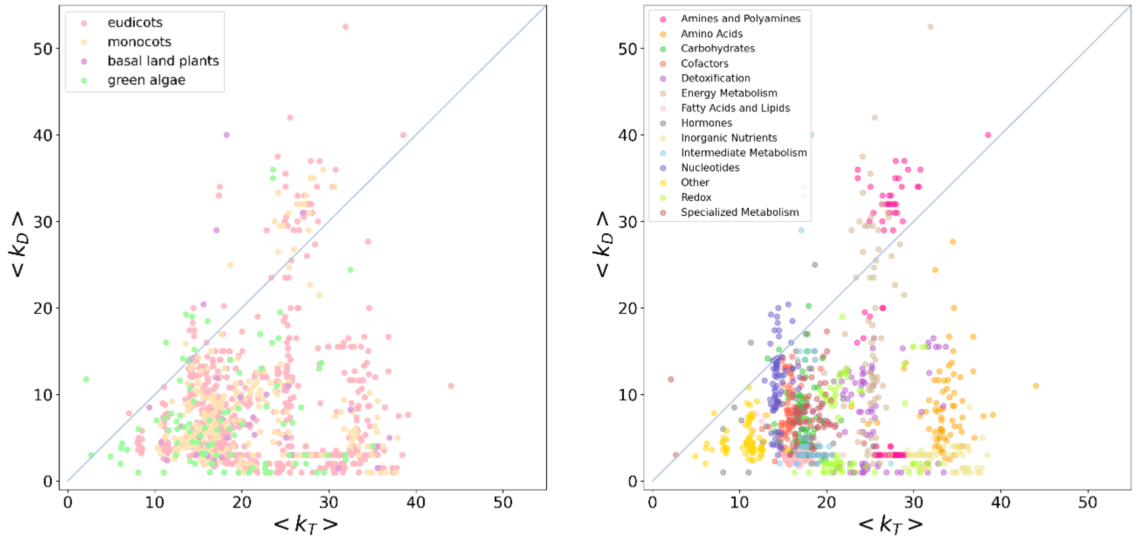

Supplementary Figure S3. (a) The mean degree of driver nodes $< k_D >$ is compared to the mean degree of functional pathways (i.e., target node sets) $<k_T>$ in the analyzed plant metabolic networks. Each dot corresponds to a pathway, and with 70 plants, we have 70 × 14 data points. The dots are colored by the four main lineage types. **(b)** The dots are colored according to the pathway functional class indicated in the legend. Similar to Fig. S2, the results show that high-degree nodes do not tend to be selected as driver nodes for target control.

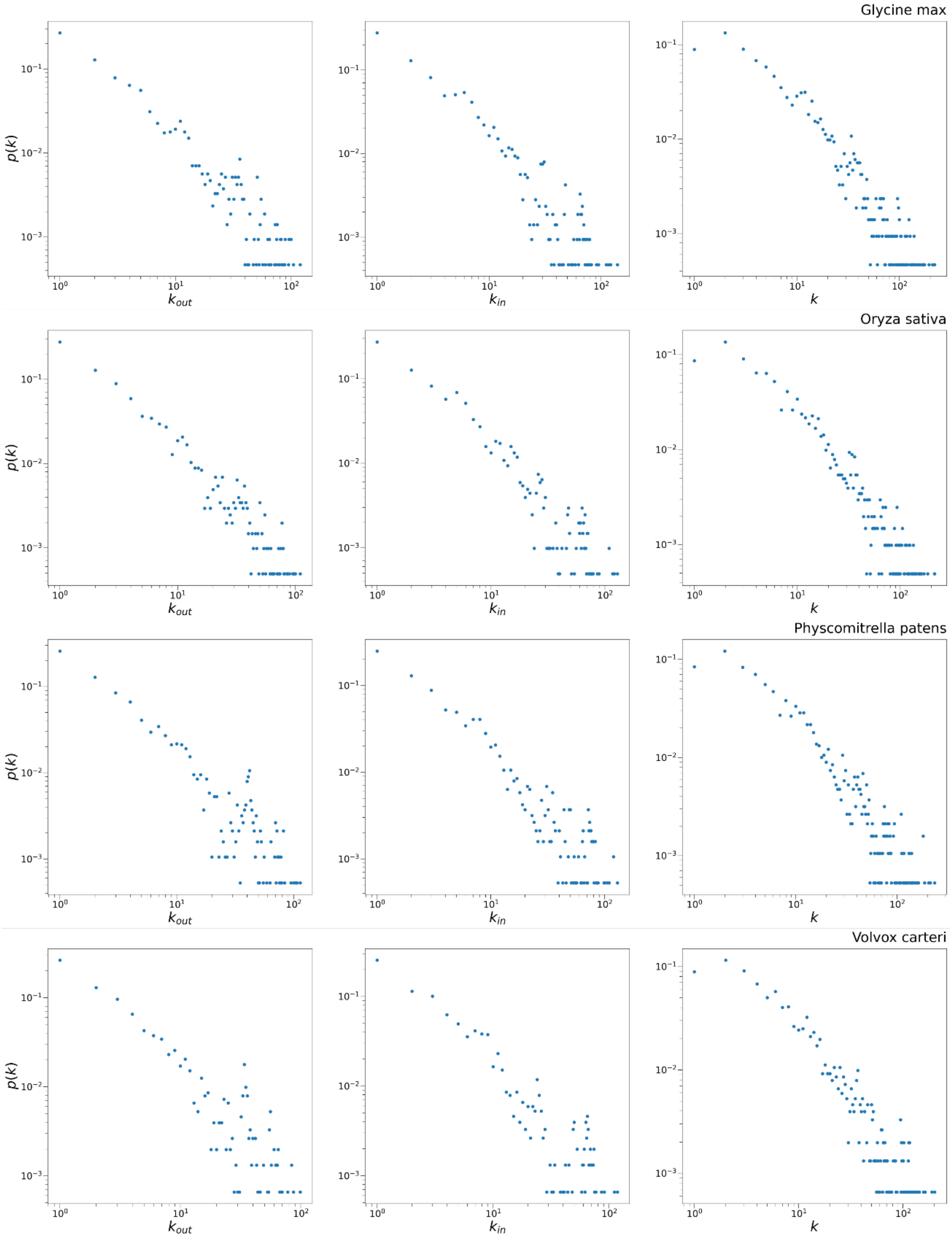

Supplementary Figure S4: The out-degree k_{out}, in-degree k_{in} and total-degree k distributions of four metabolic networks are shown. Each row corresponds to one plant metabolic network sample from the main lineages. The sample names are indicated in the figure. From top to bottom, the lineages

correspond to Eudicots, Monocots, Basal Land Plants, and Green Algae. The results show that the data consistently tends to follow a power-law distribution.

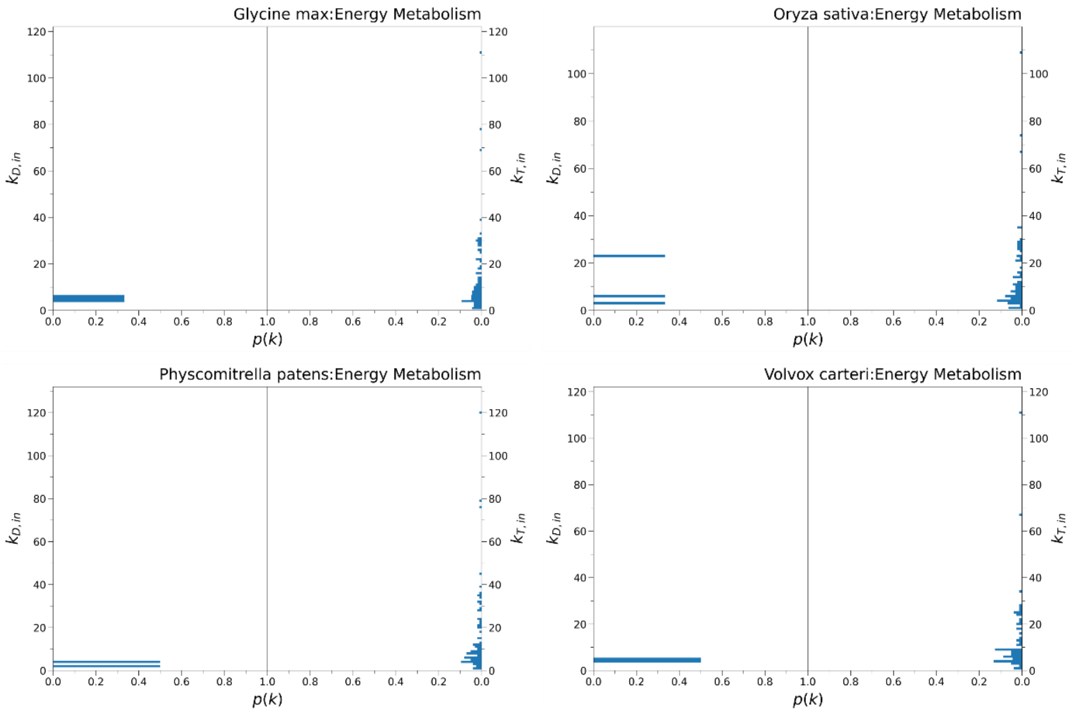

Supplementary Fig. S5. Comparisons between the in-degree distributions of the set of driver nodes (left) and the set of target nodes (energy metabolism) (right) for four metabolic networks are shown on a linear-linear scale. Each plant sample name is indicated in the figure.

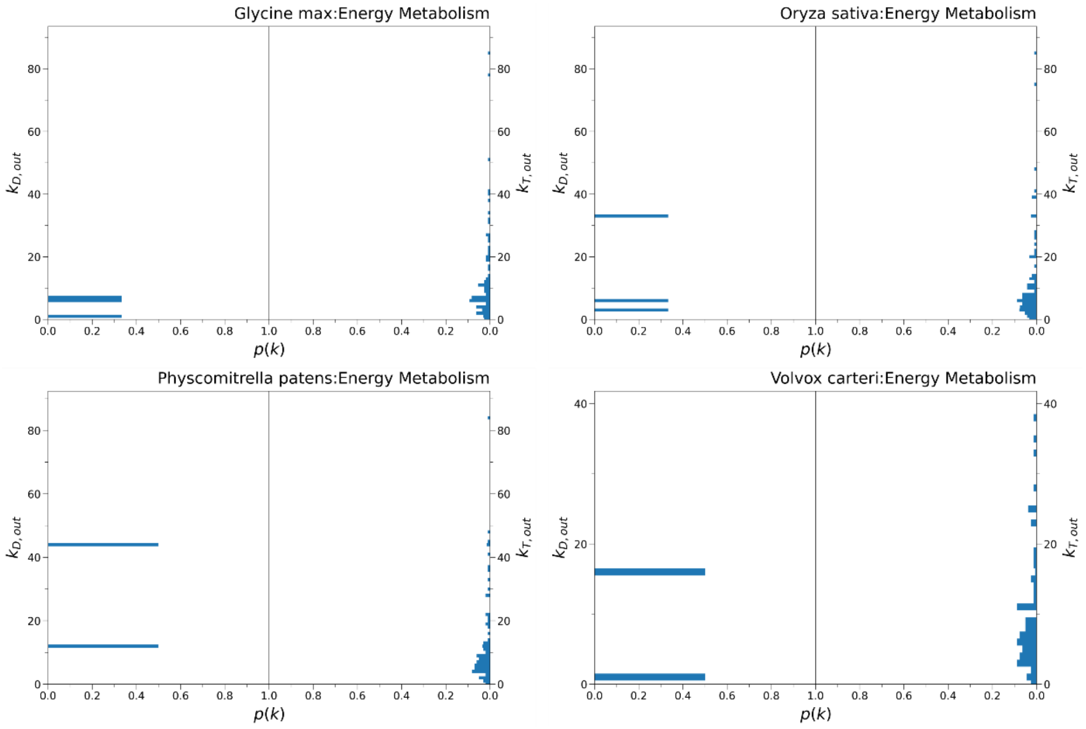

Supplementary Fig. S6. Comparisons between the out-degree distributions of the set of driver nodes (left) and the set of target nodes (energy metabolism) (right) for four metabolic networks are shown on a linear-linear scale. Each plant sample name is indicated in the figure.

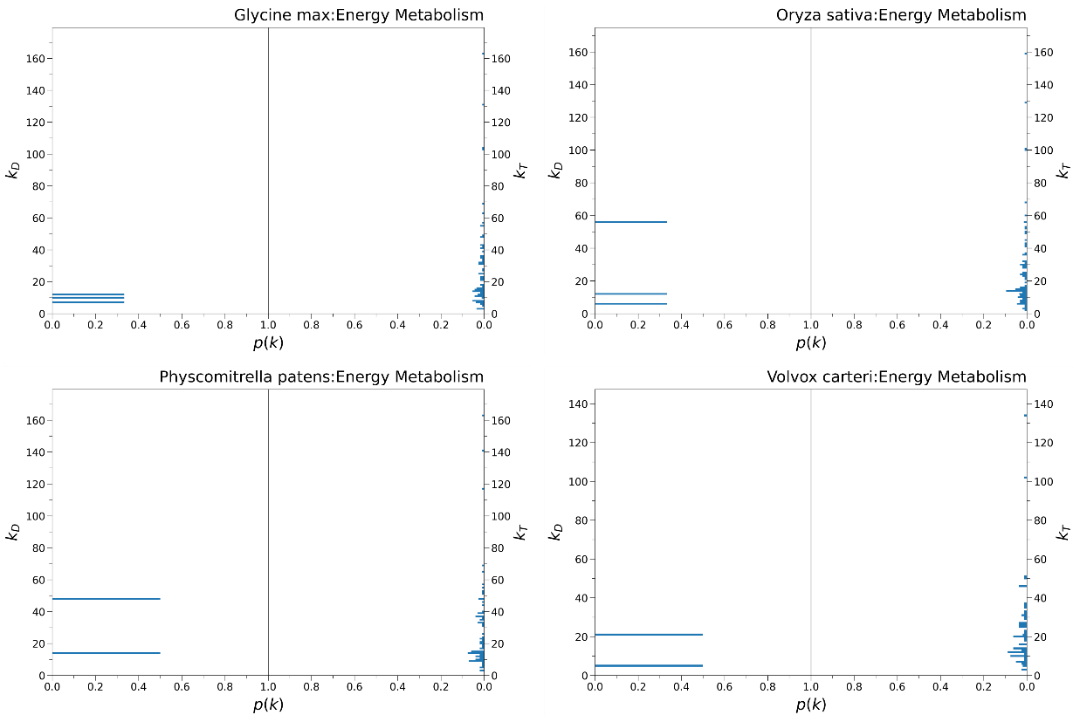

Supplementary Fig. S7. Comparisons between the total-degree distributions of the set of driver nodes (left) and the set of target nodes (energy metabolism) (right) for four metabolic networks are shown on a linear-linear scale. Each plant sample name is indicated in the figure.
